# Soybean GmDREBL Increases Lipid Content in Seeds of Transgenic *Arabidopsis*

**DOI:** 10.1038/srep34307

**Published:** 2016-10-03

**Authors:** Yu-Qin Zhang, Xiang Lu, Fei-Yi Zhao, Qing-Tian Li, Su-Ling Niu, Wei Wei, Wan-Ke Zhang, Biao Ma, Shou-Yi Chen, Jin-Song Zhang

**Affiliations:** 1 State Key Laboratory of Plant Genomics, Institute of Genetics and Developmental Biology, Chinese Academy of Sciences, Chaoyang District, Beichen West Road, Campus #1, No. 2, Beijing 100101, China; 2 School of Bioengineering & Biotechnology, Tianshui Normal University, Tianshui, Gansu 741000, China

## Abstract

A *DREB*-type transcription factor gene *GmDREBL* has been characterized for its functions in oil accumulation in seeds. The gene is specifically expressed in soybean seeds. The GmDREBL is localized in nucleus and has transcriptional activation ability. Overexpression of *GmDREBL* increased the fatty acid content in the seeds of transgenic *Arabidopsis* plants. GmDREBL can bind to the promoter region of *WRI1* to activate its expression. Several other genes in the fatty acid biosynthesis pathway were also enhanced in the *GmDREBL*-transgenic plants. The *GmDREBL* can be up-regulated by *GmABI3* and *GmABI5*. Additionally, overexpression of *GmDREBL* significantly promoted seed size in transgenic plants compared to that of WT plants. Expression of the *DREBL* is at higher level on the average in cultivated soybeans than that in wild soybeans. The promoter of the *DREBL* may have been subjected to selection during soybean domestication. Our results demonstrate that *GmDREBL* participates in the regulation of fatty acid accumulation by controlling the expression of *WRI1* and its downstream genes, and manipulation of the gene may increase the oil contents in soybean plants. Our study provides novel insights into the function of *DREB*-type transcription factors in oil accumulation in addition to their roles in stress response.

Soybean is an important economic crop and provides oil and proteins for human and animals. Increasing the FA (fatty acid) contents and improving the oil quality are closely related to our daily life. So far, numerous efforts have been made to meet the needs of human food and industry production by changing the fatty acid content in seeds^1^. However, the extracted fatty acids from the existing oil plants are far from enough, and hence traditional breeding methods and transgenic approaches manipulating fatty acid biosynthesis pathway are used to increase oil content in soybean. In plants, the pathways for lipid biosynthesis and oil accumulation had been studied and the genes related to fatty acid biosynthesis have been characterized. There are several key genes in the process of fatty acid biosynthesis. One is *ACCase* encoding acetyl CoA carboxylase in the first key step of fatty acid biosynthesis, and malonyl-CoA is produced^2^. The second one is *KASIII*, which encodes 3-ketoacyl-ACP synthase III to catalyze the formation of a 4-carbon product^3^,^4^. The carbon number of fatty acid is increased by two in acyl chain, and elongation of the acyl chain from six to 16 carbon molecules is catalyzed by an enzyme named KAS1^5^. Without KAS1, FA contents would be sharply reduced, and plant growth and development would be strongly affected^6^. The genes related to FA biosynthesis such as *Pl-PKβ1 (pyruvate kinase*), *PDHE1*α (*pyruvate dehydrogenase E1 alpha subunit*), *BCCP2 (acetyl-CoA carboxylase*), *ACP1 (acyl carrier protein*), and *KAS1* have similar expression pattern with *WRI1 (WRINKLED1*), and the FA biosynthesis-related genes were up-regulated in the *WRI1*-overexpressing plants^7^. WRI1 is an AP2-type transcription factor (TF) with two AP2 DNA-binding domains^8^, and it appears to be a master regulator of *FAS* (fatty acid synthesis) genes in expression level. There is a specific sequence motif AW-box in the promoter regions of the *FAS* genes, and WRI1 binds to this motif in *Arabidopsis*^9^. Overexpression of *WRI1* enhanced the oil content in transgenic *Arabidopsis*^6^,^8^,^9^,^10^,^11^ and maize^12^,^13^. In Castor bean, there are WRI1 binding consensus sites in the promoter region of *RcBCCP2* and *RcKAS1*, and RcWRI1 possibly binds to these sites to play a pivotal role in fatty acid biosynthesis^14^. Overexpression of a single transcription factor gene *WRI* can increase the seed oil contents while manipulating a single fatty acid biosynthesis gene had only very limited effect on the oil content^15^,^16^.

Transcription factors can regulate expression of genes involved in a wide range of plant processes and have a cascade amplification effect^8^,^17^. Therefore, transcription factors are the promising targets to improve oil contents in plants. Several candidate transcription factors involved in fatty acid biosynthesis and accumulation have been characterized, including *WRI1*^8^,^18^,^19^ and *LEC2 (leafy cotyledon2*)^20^ in *Arabidopsis. WRI1* is a target of LEC2^19^. The transcription factors regulating fatty acid contents have been identified from soybean in our lab. Two Dof-type (DNA-binding one zinc finger) genes *GmDof4* and *GmDof11* were found to increase the content of total fatty acids in their transgenic *Arabidopsis* seeds by activating the ACCase and ACSL (long-chain-acyl CoA synthetase) genes respectively^21^. Through microarray analysis, a MYB-type gene *GmMYB73* was identified and this gene can suppress expression of *GL2 (GLABRA 2*), a negative regulator of oil accumulations^22^. Overexpression of *GmMYB73* enhanced lipid contents in seeds of transgenic *Arabidopsis* through release of GL2-inhibited *PLDα1* (phospholipase D) expression^22^,^23^,^24^. Overexpression of *GmbZIP123* also enhanced lipid content and oil accumulation by regulating two sucrose transporter genes *SUC1* and *SUC5*, and three cell-wall invertase genes *cwINV1*, *cwINV3* and *cwINV6*^25^. Recently, through RNA-seq analysis, gene co-expression networks have been identified for soybean seed trait regulation and *GmNFYA (nuclear transcription factor Y alpha*) is found to enhance seed oil contents in transgenic *Arabidopsis* plants^26^.

In the present study, a DREB-type (dehydration-responsive element-binding) transcription factor gene *GmDREBL,* was cloned and found to increase the seed lipid content in the transgenic plants. GmDREBL directly activates the expression of *WRI1* to promote fatty acid accumulation. Our study provides a novel viewpoint into the regulation of the fatty acid accumulation in seeds and should add more understanding of the function for the DREB-type transcription factor gene in soybean.

## Results

### Structural features of GmDREBL and its gene expression

**Figure 1:**
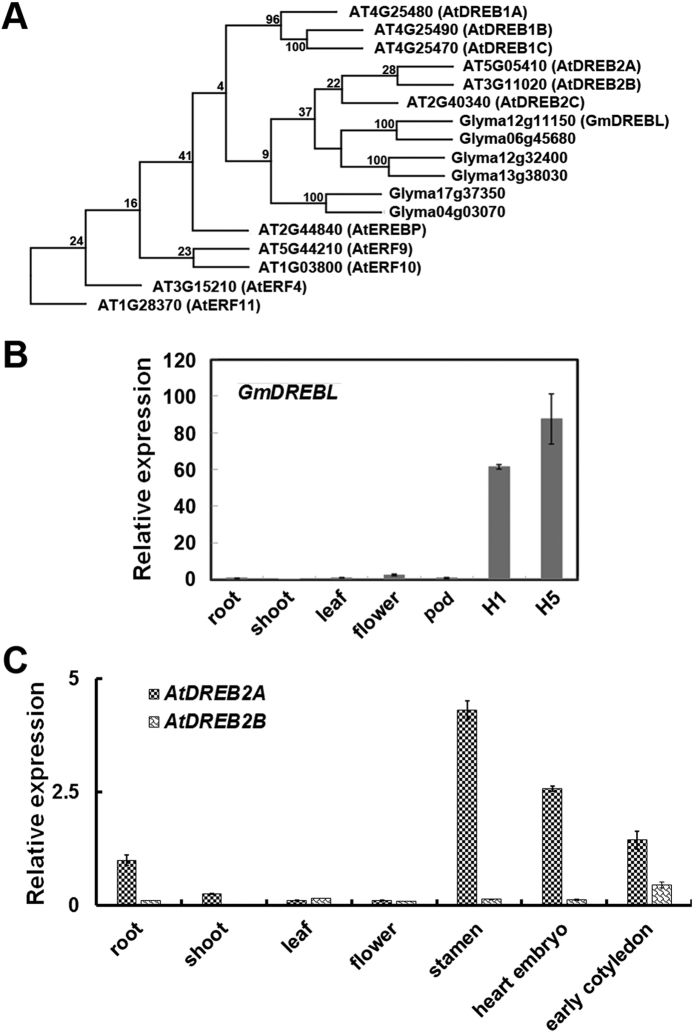
Phylogentic analysis of GmDREBL and organic-specific gene expression. (**A**) Phylogenetic tree analysis of GmDREBL protein and other DREB proteins. The phylogenetic tree was created with the high similarity sequences of AP2 domains from 6 *G. max* proteins and 11 *Arabidopsis thaliana* proteins using PHYLIP, and the bootstrap analyses were conducted with 200 replicates. The bootstrap value was showed in the branch and the length of each branch represents the distance between the sequences. (**B**) The organ-specific expression of *GmDREBL* in *G. max* cv. HN44. The roots, shoots and leaves of two weeks seedling were sampled. The pods with 2 cm length and the opening flowers of adult plant were sampled. Two developmental stages of soybean seeds were selected. Weight of a H1 developing seed is only 4% of highest seed fresh weight. Weight of a H5 developing seed is 35% of highest seed fresh weight. (**C**) The organ-specific expression of *AtDREB2A* and *AtDREB2B in Arabidopsis thaliana*. The homologues of GmDREBL with high identity in *Arabidopsis* were AtDREB2A and AtDREB2B (e-value < 1.3E-30). Their expression was evaluated by publicly available data of microarray in AtGenExpress of TAIR (http://www.arabidopsis.org).

Using high-throughput RNA-seq analysis, 87 transcription factor genes have been identified in developing seeds along with the fatty acid accumulation^25^. These genes were tested for lipid regulation through transgenic analysis. Among these, one gene encoding an AP2 domain protein of 211 amino acids was further studied. Because the encoded protein was clustered with the DREB subfamily of AP2 family (Fig. 1A), the gene was named as *GmDREBL (Glyma12g11150*).

The expression of *GmDREBL* was examined in different organs of soybean plants. The gene was highly expressed in the H1 and H5 stages of the developing seeds in comparison with the other organs tested (Fig. 1B). The homologues of *GmDREBL* in *Arabidopsis* were *AtDREB2A (AT5G05410*) and *AtDREB2B (AT3G1102*0) (e-vlaue < 1.3E-30). Both of them exhibited high expression in the developing seeds (heart embryo and early cotyledon stages), in addition that *AtDREB2A* also had high expression in root and stamen of *Arabidopsis* (Fig. 1C). These results suggest that *GmDREBL* may be involved in regulation of the seed-related process.

### GmDREBL subcellular localization and transcriptional activation

**Figure 2:**
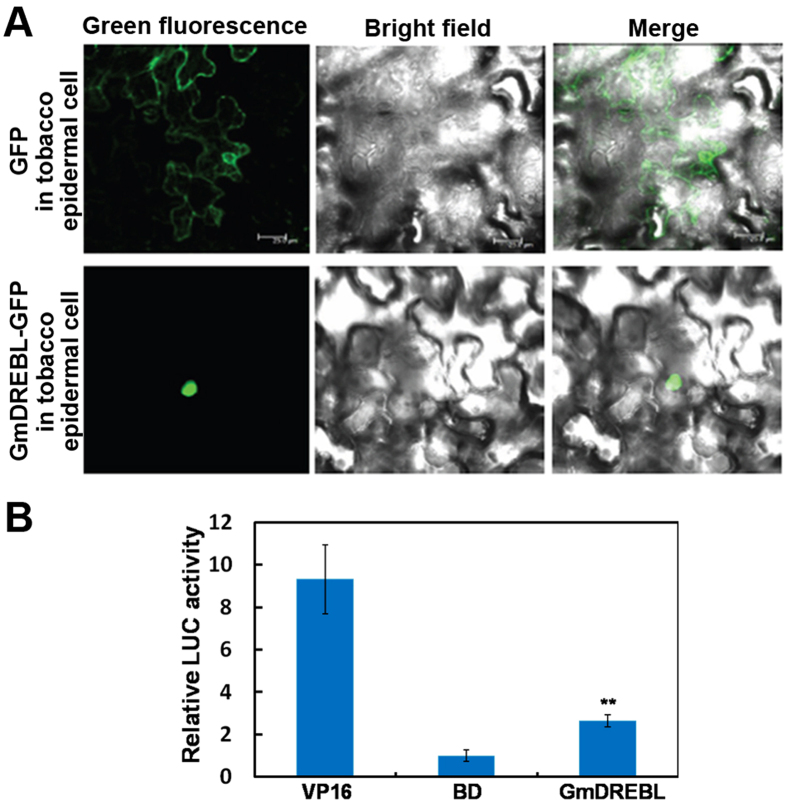
Subcellular localization and transactivation activity of GmDREBL. (**A**) Subcellular localization of GmDREBL in leaf epidermal cells of tobacco by transient expression assay. (**B**) Transactivation activity analysis of GmDREBL in *Arabidopsis* protoplasts. Bars indicate SD (n = 4). **Indicate significant difference compared to BD (P < 0.01).

The *GmDREBL* encoded a putative DREB/AP2-type transcription factor. We then examined the subcellular localization of GmDREBL by transiently expressing the gene in epidermal cells of tobacco leaves. The lower panel of Fig. 2A showed that GmDREBL was located in the nucleus, while the upper panel of Fig. 2A showed that GFP control was mainly observed in the cytoplasm.

The transcription activation ability of GmDREBL was further examined by using a dual-luciferase reporter (DLR) assay system in *Arabidopsis* protoplasts. The coding sequence of *GmDREBL* and the DNA sequence encoding the GAL4 DNA-binding domain (GAL4DBD) were combined to generate pBD-GmDREBL effector plasmid. The vector containing only the *GAL4DBD* sequence was used as a negative control and the vector fused with *VP16* was used as a positive control. As shown in Fig. 2B, GmDREBL activated the reporter *LUC* gene, compared to the negative control BD, indicating that GmDREBL has the transcriptional activation activity. Given that the GmDREBL is located in the nucleus and has transcriptional activation activity, this protein is likely a transcription factor.

### *GmDREBL* increased the fatty acid content in seeds of transgenic plants

**Figure 3:**
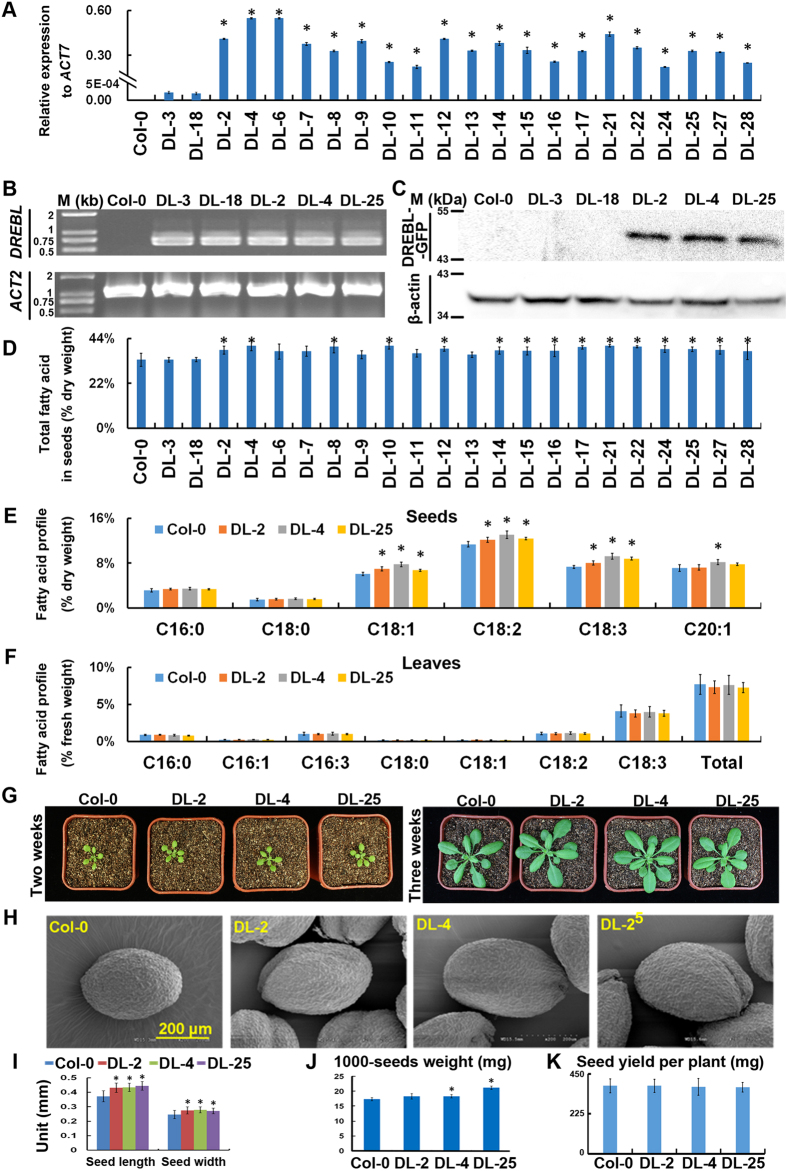
The effect of *GmDREBL* on seed-related traits. (**A**) Expression of *GmDREBL* in transgenic plants. The relative expression level of *GmDREBL* was normalized using *AtACTIN7* as an internal control. Bars indicate SD (n = 4). (**B**) Examination of *GmDREBL* presence by PCR in transgenic plant using genomic DNA as a template. An actin 2 gene was also amplified as the control. (**C**) Detection of GmDREBL-GFP in transgenic plants by Western blot analysis using mouse anti-GFP antibody. And the detection of β actin was used as the control. (**D**) Overexpression of *GmDREBL* increased total fatty acid content of transgenic *Arabidopsis* seeds. Bars indicate SD (n = 4). (**E**) The profile of fatty acids in dry seeds of Col-0 and three lines DL-2, DL-4 and DL-25. Bars indicate SD (n = 4). (**F**) The profile of fatty acids in fresh leaves of 12-day-old seedlings from Col-0 and three lines DL-2, DL-4 and DL-25. Bars indicate SD (n = 4). (**G**) The rosette phenotypes of Col-0 and three homozygous lines DL-2, DL-4 and DL-25 in two and three weeks. (**H**) The scanning electron micrograph of the seed coat of Col-0 and transgenic plants with *GmDREBL*. The white line stands for 200 μm. (**G**) The statistical result of seed length and seed width of wild type Col-0 plant and transgenic plants with *GmDREBL*. Bars indicate SD (n = 12). (**I**) Overexpression of *GmDREBL* increased 1000-seed weight of transgenic *Arabidopsis* seeds. Bars indicate SD (n = 4). (**J**) The seed yield per plant of Col-0 and three homozygous lines DL-2, DL-4 and DL-25. Bars indicate SD (n = 10). All the asterisks indicate significant difference compared to the corresponding controls (p-value < 0.05).

To investigate the function of *GmDREBL* during the plant growth and development, the *GmDREBL* was fused with a *GFP* gene, driven by 35S promoter and transformed into *Arabidopsis* plants, and the *GmDREBL*-overexpressing lines were identified by qRT-PCR. We totally screened 22 transgenic lines and most of these lines showed relatively higher levels of *GmDREBL* expression compared to the Col-0 (Fig. 3A). However, two lines (DL-3 and DL-18) exhibited extremely low expression level (Fig. 3A–C), and may be regarded as vector control for comparison.

Because the RNA-seq-identified transcription factors are roughly correlated with fatty acid accumulation during soybean seed development^25^, we examined whether the *GmDREBL* alters the fatty acid accumulation in the overexpressing transgenic lines. Among these lines, 15 overexpression lines exhibited significantly higher total fatty acid content in seeds than vector control (DL-3 and DL-18) and Col-0 (Fig. 3D). The left lines also showed increases in seed total fatty acid contents on average. This result suggests that *GmDREBL* has a positive role on total fatty acid accumulation in seeds.

To further decipher which composition of fatty acid could be regulated by *GmDREBL*, we selected three homozygous T3 transgenic lines DL-2, DL-4 and DL-25 for further analysis. The profile and content of fatty acids in the seeds of Col-0 and the three lines were compared. The result showed that overexpression of *GmDREBL* significantly enhanced the contents of C18:1, C18:2, C18:3 in all the three transgenic lines and the C20:1 content in DL-4 overexpression lines (Fig. 3E). The profile and content of fatty acid were also measured in leaves of the transgenic plants and Col-0 plants, and no significant difference was observed between transgenic lines and Col-0 (Fig. 3F). These results indicate that *GmDREBL* promotes accumulation of fatty acids specifically in seeds, but not in leaves of the transgenic plants.

### Overexpression of *GmDREBL* enhances seed size in transgenic *Arabidopsis*

We further examined the effects of *GmDREBL* overexpression on seedling growth and other seed-related traits. We found that there was no difference in rosette size between overexpression lines and Col-0 at different stages of seedling growth (Fig. 3G). The seed size and the 1000 seed-weight were substantially greater in the overexpressing lines than that in Col-0 (Fig. 3H–J). The GmDREBL-GFP protein can be detected in the seed integument cells (Figure S1A). However, the seed yield per plant did not change (Fig. 3K). The length of silique in the three transgenic lines was not significantly different from that in Col-0 either (Figure S1B,C). These results suggest that the *GmDREBL* increases the seed size/weight but not the total seed yield per plant in transgenic *Arabidopsis*.

### GmDREBL activates expression *of WRI1* and other fatty acid biosynthesis-related genes

**Figure 4:**
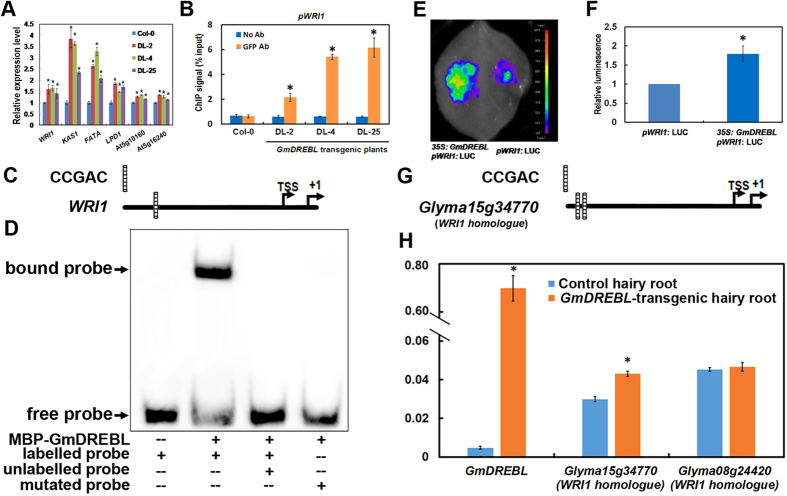
GmDREBL can regulate the expression of *WRI1.* (**A**) The expression level of WRI1 and its downstream genes in Col-0 plants and GmDREBL-overexpressing plants. *Indicate significant difference (p-value < 0.05). (**B**) The intensity of ChIP signal of Col-0 plants and GmDREBL-overexpressing plants with a GFP-tag for GmDREBL. The WRI1 gene promoter is enriched in GmDREBL-overexpressing plants. Bars indicate SD (n = 4). *Indicates significant difference (P < 0.05). (**C**) The LUC image of tobacco leaves co-infiltrated with *A. tumefaciens* GV3101 containing different combination of constructs. (**D**) Quantitative analysis of LUC luminescence intensity in (**C**). Bars indicate SD (n = 7). *Indicates significant difference (p-value < 0.05). (**E**) The DRE cis-element present in *WRI1* promoter sequence. (**F**) Gel-shift assay showing that GmDREBL can bind the promoter of *WRI1*. 100-fold unlabeled probe was used in the third lane. CCGAC motif in Labelled mutant probe was changed to TTATT in fourth lane. Arrows indicate the position of the protein–DNA complex. (**G**) Illustration of CCGAC motif distribution in the promoters of *Glyma15g34770 (WRI1 homologue,* e-value = 3.4E-101). (**H**) *GmDREBL* enhanced the expression of *Glyma15g34770* in *GmDREBL-*transgenic hairy root of soybean, but not the expression of *Glyma08g24420 (WRI1 homologue,* e-value = 3.4E-101). *Indicate significant difference (p-value < 0.05).

As the total fatty acids (FA) showed a substantial increase in the seeds of the *GmDREBL-*overexpressing transgenic plants, it is possible that the GmDREBL regulates the expression of genes involved in FA biosynthesis. We then examined the expression levels of 13 FA-biosynthesis related genes including *NAD(P)-binding Rossmann-fold superfamily (At01g24360*, *At3g03980*, *At4g13180*, *At5g18210*), *KASIII (At01g62640*), *FAD1 (At01g74960*), *Beta-ketoacyl synthase (At02g04540*), *Enoyl-ACP reductase 1 (At02g05990*), *Thioesterase superfamily (At02g22230, At5g10160*), *EMB3147 (At02g30200*), *aldehyde reductase (At3g04000*), and *KASI (At5g46290*). Among these, the gene encoding KASI and the gene (*At5g10160*) encoding a thioesterase superfamily protein were upregulated in the *GmDREBL-*overexpressing lines (Fig. 4A). Because these two genes can be directly regulated by WRI1, a key regulator in the biosynthesis of fatty acids^8^,^9^,^10^,^19^, we further tested the *WRI1* expression by qRT-PCR. We find that overexpression of *GmDREBL* significantly up-regulated *WRI1* expression (Fig. 4A). The WRI1 downstream genes *FATA*, *LPD1*, and At5g16240 (plant stearoyl-acyl-carrier-protein desaturase family protein) were also enhanced in transgenic plants (Fig. 4A). These results indicate that the GmDREBL promotes expression of *WRI1* and other fatty acid biosynthesis-related genes.

We further detected whether GmDREBL can directly bind to the promoter of *WRI1* by ChIP-qPCR and gel shift assay. Figure 4B showed that, GmDREBL protein expressed in the siliques of the *GmDREBL-GFP* transgenic plants significantly enriched the promoter region of *WRI1*, as revealed by anti-GFP antibody. In contrast, the sample of Col-0 plants without GmDREBL protein rarely enriched the *WRI1* promoter region. The promoters of *WRI1* contain one DRE cis-element of CCGAC (Fig. 4C). We next asked whether GmDREBL could directly bind to the promoters of *WRI1*. The fragment covering CCGAC motifs and flanking sequence was identified as candidate binding sites in *WRI1* promoter. GmDREBL was found to specifically bind to the fragment (Fig. 4D, second and third lane from left). However, GmDREBL could not bind to the mutated version of the fragments (Fig. 4D, fourth lane from left). These results demonstrate that GmDREBL can directly bind to the promoter of *WRI1* in plants.

We then examined whether the *WRI1* promoter activity was enhanced by GmDREBL using the luciferase expression system *in vivo* by transiently expressing the constructs in tobacco leaf. The *WRI1* promoter: LUC reporter construct (*pWRI1:*LUC) in pGWB435 or pGWB405-*GmDREBL* construct (35S:GmDREBL) was transfected into agrobacterium. After culture in LB liquid medium at 28°C overnight, the agrobacteria were collected and suspended in the infiltration buffer. The two transfected agrobacteria were injected into the tobacco leaves solely or together, and cultured for 3 days. After that, the leaves were detected by a low-light cooled charge-coupled device imaging apparatus, and we find that the GmDREBL enhanced the LUC activity driven by *WRI1* promoter (Fig. 4E,F). These results imply that the GmDREBL can stimulate *WRI1* promoter activity.

To further confirm that GmDREBL could enhance the expression of *WRI1* homologue in soybean. We identified two homologues of *WRI1* in soybean, namely *Glyma15g34770* (e-value = 3.4E-101) and *Glyma08g24420* (e-value = 3.4E-101). We found that two CCGAC cis-elements are present in the promoter of *Glyma15g34770* (Fig. 4G). By overexpression of *GmDREBL* in soybean transgenic hairy roots, we found that GmDREBL could enhance the expression of *Glyma15g34770* but not the *Glyma08g24420* (Fig. 4H).

### *GmDREBL* is upregulated by GmABI3 and GmABI5

**Figure 5:**
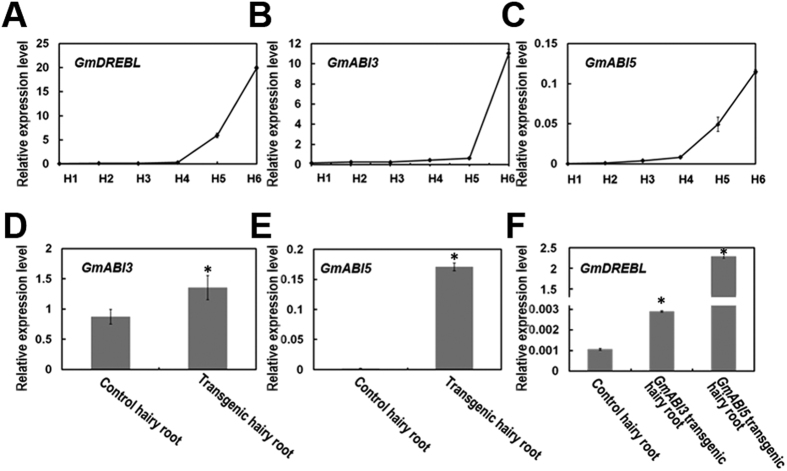
*GmDREBL* is up-regulated by GmABI3 and GmABI5. (**A**) The expression level of *GmDREBL* in different stages of seed development. Six developmental stages of soybean seeds were selected according to the ratio of seed weight of the stage to highest seed fresh weight, namely 4, 8, 12, 20, 35, and 80% (H1–H6). (**B**) The expression level of *GmABI3* in different stages of seed development. (**C**) The expression level of *GmABI5* in different stages of seed development. (**D**) The expression of *GmABI3* in soybean transgenic hairy roots harboring *GmABI3*. (**E**) The expression of *GmABI5* in soybean transgenic hairy roots harboring *GmABI5*. (**F**) The expression of *GmDREBL* in soybean transgenic hairy roots expressing *GmABI3* or *GmABI5*. Bars indicate SD (n = 4). All the asterisks indicate significant difference compared to the corresponding controls (p-value < 0.05).

*Arabidopsis* ABI3 and ABI5 are key regulators for fatty acid accumulation^21^,^27^, and ABI3 contains AP2 domain whereas ABI5 belongs to bZIP family. There are putative *cis*-elements in the promoter region of *GmDREBL* for possible AP2-domain protein and bZIP protein binding. We then determined if *GmDREBL* has any co-expressions with *GmABI3 (Glyma08g47240*) and *GmABI5 (Glyma10g08370*) from soybean, whose identity with ABI3 and ABI5 is 71.7% and 81.3%, respectively. The expression pattern of *GmABI3*, *GmABI5* and *GmDREBL* was determined during soybean seed development by real-time PCR, and these genes have similar expression pattern (Fig. 5A–C), consistent with the accumulation trend of fatty acids in our previous report^25^. These results imply that *GmDREBL* may be regulated by GmABI3 and GmABI5.

We further constructed pBI121-*GmABI3* and pBI121-*GmABI5* vectors, and transfected them into *Agrobacterium rhizogenes* K599. These agrobacteria were used to infect hypocotyls of soybean seedlings for generation of transgenic hairy roots with overexpression of *GmABI3* or *GmABI5* (Fig. 5D,E). The expression of *GmDREBL* was increased in the transgenic hairy roots overexpressing *GmABI3* or *GmABI5* (Fig. 5F). These results suggest that GmABI3 and GmABI5 promote *GmDREBL* expression.

### *GmDREBL* is subjected to selection during domestication

**Figure 6:**
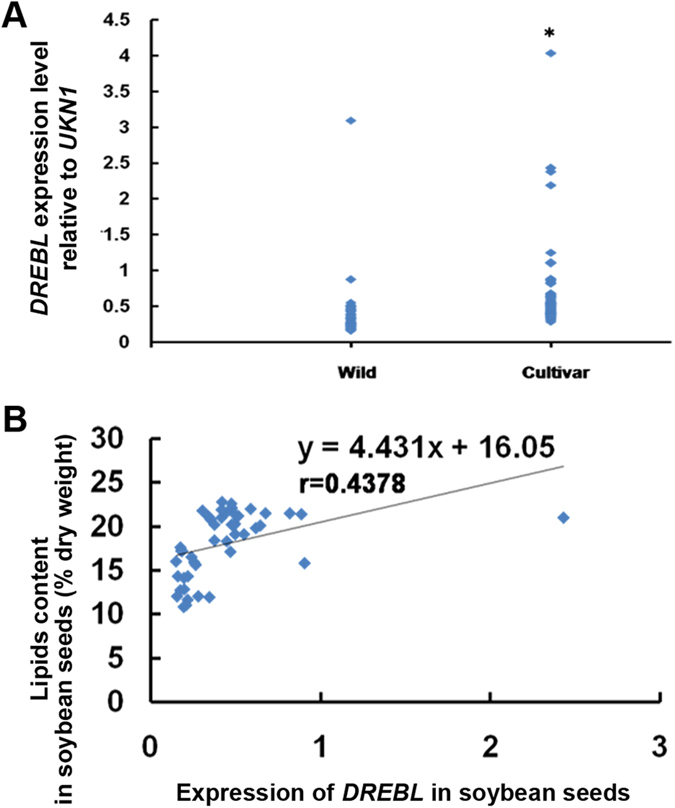
*DREBL* expression in wild and cultivated soybeans. (**A**) The expression level of *DREBL* in different wild type and cultivated soybeans. Asterisks indicate significant differences compared with the Col-0 plants (P < 0.05). (**B**) Correlation between expression of *DREBL* and lipid content in soybean seeds. r = 0.4278.

Our above study indicates that overexpression of *GmDREBL* promotes accumulation of fatty acid content in seeds of transgenic plants (Fig. 3). Considering that cultivated soybeans usually had much higher oil contents compared to the wild soybeans, we investigated whether there is any difference in the expression levels of *GmDREBL* from cultivated soybean and *GsDREBL* from wild type soybean. It can be seen that the average expression level of *GmDREBL* in cultivated soybeans is significantly higher than that of *GsDREBL* in wild soybeans (Fig. 6A), suggesting that the *DREBL* expression may have been subjected to selection during domestication of soybean. We further determined the relationship between expression levels of *DREBL* and seed oil contents in 76 wild and cultivated soybeans, and found that the seed oil contents were positively correlated with the *DREBL* gene expressions with a coefficiency of 0.4378 (Fig. 6B).

**Figure 7:**
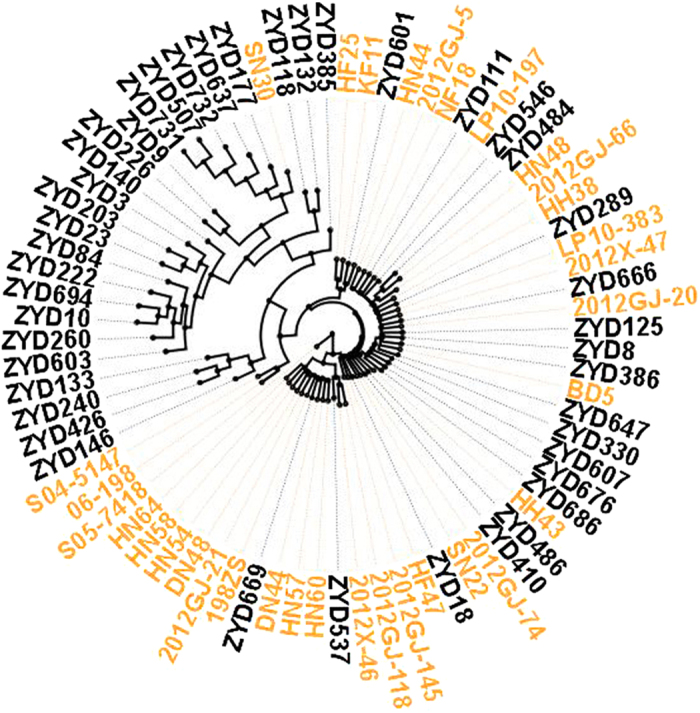
Phylogenetic tree analysis of *DREBL* promoter in different wild type and cultivated soybeans. Phylogenetic tree of *DREBL* promoter sequences from 33 cultivated soybeans and 43 wild soybeans. The yellow names indicated the cultivated soybean accessions and the black names indicated the wild soybean accessions. The tree was constructed by using PHYLIP, and the bootstrap analyses were conducted with 1000 replicates.

Since the *DREBL* gene expression is relatively higher in the cultivated soybeans, and the oil contents are roughly correlated with the *DREBL* gene expression levels among all the soybeans tested, we determined the sequences of *DREBL* promoter regions to see if there is any natural variations. The promoters of *GmDREBL* from 33 cultivated varieties and *GsDREBL* from 43 wild type soybeans were cloned and sequenced. The phylogenetic tree analysis was performed and we found that most of the wild soybeans were clustered together (Fig. 7, names in black), however, some of the wild soybeans clustered with cultivated soybeans (Fig. 7, names in yellow), possibly suggesting a close relationship of the cultivated soybeans with the few wild soybeans in the same group.

We further compared the nucleotide diversity among the promoter sequences and found 7 major insertion/deletion (indel) regions (Table 1). Based on the combination of these indels, seven major haplotypes were identified (Table 1). Type 1 had only one cultivar with a specific 15 bp-insertion at −944 position in the *DREBL* promoter. Type 2 had 19 wild soybeans and 32 cultivated soybeans, and Williams 82 belonged to this type 2. Type 1 and 2 had higher oil contents than the Type 3 to 7, and Type 3 to 7 all had wild soybeans (Table 1, Table S1). Type 2 haplotype is very similar to the Type 7, and Type 7 had two more ‘T’ than Type 2. These results indicate that specific indel combinations in cultivated and/or wild soybeans may contribute to the increase of oil contents. It should be noted that the coding sequences of the *DREBL* gene in the wild and cultivated soybeans did not show much variations^28^.

We calculated the π value representing the genetic diversity of these promoter sequences and found that wild soybeans had a π value of 0.012299, whereas the cultivated soybeans had a much lower π value of 0.002058, indicating that the wild soybeans have a relatively higher level of genetic diversity in the *DREBL* gene. These results suggest that the cultivated soybeans may have been selected in the *DREBL* gene promoters during domestication.

## Discussion

Our previous studies have demonstrated that transcription factors GmDof4, GmDof11, GmMYB73, GmbZIP123 and GmNFYA play important roles in regulation of seed-related traits and fatty acid biosynthesis^22^,^25^,^26^,^27^. Presently, we further found that an AP2-domain protein GmDREBL promotes fatty acid accumulation in seeds of the transgenic *Arabidopsis* through up-regulation of the master regulatory gene *WRI1* and other genes related to fatty acid biosynthesis. The GmDREBL also enhances seed size and seed weight. The expression of the *DREBL* gene is relatively higher in developing seeds of the cultivated soybeans compared to that in wild soybeans, and the expression level is roughly positively correlated with the oil contents in all the soybean accessions. The promoter sequences were further analyzed and the genetic variations in these sequences showed correlations with fatty acid accumulation in soybean seeds.

GmDREBL is located in nucleus and has obvious transcriptional activation ability (Fig. 2). These two features, together with the finding that GmDREBL binds to *WRI1* gene promoter and activates this promoter activity (Fig. 4), demonstrate that GmDREBL is a transcription factor directly regulating *WRI1* gene expression. In the gene co-expression network study, Glyma15g34770 (WRI1 homologue) was found to be co-expressed with the GmDREBL (Glyma12g11150)^29^. We found that GmDREBL could enhance the expression of Glyma15g34770 in soybean transgenic hairy root. This suggests that they may participate in the regulatory network for TAG synthesis. Considering that the *GmDREBL* is abundantly expressed in developing seeds (Fig. 1), and WRIl is a master regulator of lipid biosynthesis, it is most likely that the GmDREBL would activate *WRI1* for lipid accumulation especially in seeds. Consistent with this, the lipid contents in leaves of the *GmDREBL*-overexpressing transgenic plants was not enhanced, probably due to that the gene is barely expressed in leaves (Fig. 1). The GmDREBL localization was also examined in integument cells of *GmDREBL-GFP* transgenic *Arabidopsis* seeds, and the GmDREBL-GFP seemed to be localized in nucleus and some other regions of the cells (Figure S1A). The localization of the GmDREBL in regions other than the nucleus is probably due to the constitutive expression of the gene driven by the 35S promoter.

Genes in *de novo* synthesis of FAs was further examined and the expression of *KAS1* (b-Ketoacyl-[acylcarrier protein] synthase I) was significantly increased in *GmDREBL*-overexpressing transgenic lines compared to WT plants (Fig. 4A). KAS1 is responsible for the elongation of fatty acid (FA) synthesis from C4 to C16^6^. Lacking of *KAS1* leads to an obvious change in the polar lipid component, abnormal embryo development before the globular stage, and sharp decrease in FA levels in seeds. Genes such as *Pl-PKb1*, *PDHE1a*, *FAD2*, *FAD3*, *BCCP2*, *ACP1*, *oleosin*, and *KAS1* in fatty acid synthesis have similar expression patterns with *WRI1* during the seed developmental process^7^. These gene expressions increased in *WRI1*-overexpressing plants but decreased in mutants with down-regulated *WRI1* expression. WRI1 is a key regulator in FA biosynthetic pathway. Two BnWRI1 genes, *BnWRI1-1* and *BnWRI1-2,* promoted seed oil increase by 10–40% in transgenic plants overexpressing *BnWRI1-1* or *BnWRI1-2*, and the seed size was also enlarged^11^. Overexpression of *BnSTM* resulted in the induction of genes relevant to FA synthesis including *BnLEC1*, *BnLEC2* and *BnWRI1*, and seed oil content was also increased in the transgenic plants^30^. In *Arabidopsis*, WRI1 regulated the expression of *BCCP2*^19^. BCCP2 is involved in fatty acid biosynthetic process and has acetyl-CoA carboxylase activity. *ZmWRI1a*, the homologue of *AtWRI1* in maize, complemented the reduced fatty acid content of *Atwri1-4* mutant, and overexpression of *ZmWRI1a* dramatically increased the fatty acid content in maize grain^13^. Studies also reported that overexpression of *ZmWRI1* resulted in an oil increase^12^. In the present study, we proved that *AtWRI1* or its soybean homologue was upregulated in transgenic plants/transgenic hairy roots overexpressing *GmDREBL* (Fig. 4A,H). ChIP-qPCR, EMSA and transient transcriptional activation experiments in tobacco leaves (Fig. 4B–F) further demonstrate that GmDREBL can directly bind to the promoter and activate *WRI1* expressions, finally leading to FA synthesis and oil accumulation.

It should be noted that there is an ABA-responsive elements (ABRE) in the promoter of *GmDREBL*, and ABI3 and ABI5 are also master regulatory factors for fatty acid biosynthesis^21^,^31^. The expression of *GmABI3*, *GmABI5*, *GmDREBL* and *GmWRI1* shared the same expression pattern and was in accordance with the fatty acid accumulation trend (Fig. 5). Through soybean-transgenic hairy root experiments, we proved that *GmDREBL* can be upregulated by GmABI3 and GmABI5. These results indicate that GmABI3 and GmABI5 may be the upstream regulator of *GmDREBL*.

Because the average *DREBL* expression level in cultivated soybeans is significantly higher than that in wild soybean plants (Fig. 6A), we compared the promoter regions of the *DREBL* in the wild and cultivated plants. We identified at least seven haplotypes of *DREBL* promoters, and compared to type 2 containing the reference Williams 82 soybean, the type 1 has two major insertions at −404 and −944 bp positions (Table 1). The type I contains only one cultivar SN30 with high oil content (Table S1), which is clustered with many wild soybeans (Fig. 7). This *DREBL* allele from SN30 may be further tested in breeding for potential improvement or alteration of oil content. Alternatively, the type 2 allele, which is prevalent in many cultivars, may be introduced into SN30 for further improvement of oil accumulation.

DREB-like transcription factors are generally stress-related and could be induced by abiotic stresses such as drought and high salt^32^,^33^,^34^. We also found that *GmDREBL* could be sharply induced by drought and high salt (Figure S2A,B), and overexpressing *GmDREBL* increased the survival rate of transgenic plants under 200 mM NaCl treatment (Figure S2C,D). The GmDREBL could directly bind to the promoter region of stress-related gene *LEA* and activate *LEA* expression for stress tolerance (Figure S3). The GmDREBL also promoted expression of the *AtDREB2B (AT3G1102*0) (Figure S1D), which may further contribute to stress tolerance of the transgenic plants.

In conclusion, overexpressing *GmDREBL* increased fatty acid accumulation in seeds of transgenic plants, likely through the control of a key regulator *WRI1* for FA synthesis. Besides, *GmDREBL* can be regulated by GmABI3 and GmABI5. Our study provides novel insight to the function of *DREB-type* genes in both oil accumulation and seed size control in addition to their roles in stress response. Further manipulation of the *GmDREBL* in soybean and evaluation in field test should shed light on its roles in regulation of oil contents in seeds.

## Methods

### Plant materials and growth conditions

Soybean (*G. max*) seeds of cultivar Heinong44 were planted in the experimental farm of IGDB, CAS, in Beijing. *Arabidopsis* plants were grown at 22 °C with a photoperiod of 16 h/8 h (light/dark) per day. All plant materials were harvested and stored at −70 °C for RNA isolation.

### Cloning of the *GmDREBL* gene

The full-length opening reading frame of *GmDREBL* was amplified from Heinong44 leaf RNA, and cloned into the Gateway-T vector (invitrogen, pCR8/GW/TOPO TA Cloning Kit). This construct was confirmed by sequencing. PCR primers were listed in Table S2.

### Plasmid construction and protein subcellular localization in Tobacco leaves

35S-GmDREBL-GFP was generated using vector pGWB405 from invitrogen driven by 35S promoter. As *GmDREBL* has already been cloned into T-vector, the *GmDREBL* gene was further cloned into the pGWB405 vector through gene recombination, with GFP fusion at the downstream. The pGWB405 vector containing the GFP gene driven by the 35S promoter was used as a control. Each plasmid was transformed into *Agrobacterium tumefaciens* GV3101. The *Agrobacterium* strain was incubated in LB medium overnight and re-suspended in infiltration buffer (100 μM acetosyringone, 10 mM MES and 10 mM MgCl2) to an ultimate concentration of 1.0_OD=600_. And then the strain was infiltrated into tobacco leaves. The infiltrated plant leaves were incubated in greenhouse for 3 days, and the subcellular localization of the GmDREBL protein was visualized under a confocal microscope (Leica TCS SP5).

### Transcriptional activation analysis in *Arabidopsis* protoplasts

The transcriptional activation activity was examined in the *Arabidopsis* protoplast system performed as described previously^35^. The reporter was a plasmid generated from pUC19 containing the firefly LUC reporter gene, and was driven by a modified 35S promoter plus 5 × UAS (upstream activating sequence)^36^. The *GmDREBL* gene was fused into the GAL4 DNA BD-coding sequence and constructed into pRT107 to generate an effector plasmid pRT-BD-GmDREBL under the control of 35S promoter. pRT107 vector containing the BD sequence was used as a negative control and the vector containing the BD-VP16 fusion sequence as a positive control. A pPTRL plasmid containing a CaMV 35S promoter and *Renilla* LUC was used as an internal control. The constructs above were transformed into *Arabidopsis* protoplasts by PEG-mediated transfection. Using a GloMaxTM 20/20 Luminometer (Promega), luciferase activity was measured for each co-transfection sample after culturing for 16–20 h.

### Generation of *GmDREBL* transgenic *Arabidopsis* plants

The pGWB405-*GmDREBL-GFP* vector was transfected into *A. tumefaciens* GV3101 by electroporation, then introduced into *Arabidopsis* Col-0 plants by the vacuum infiltration method^37^. Homozygous transgenic lines were obtained and the lines DL-2, DL-4 and DL-25 with different expression levels were selected for further analysis. The subcellular localization of the GmDREBL protein in integument cells of seed was visualized under a confocal microscope (Leica TCS SP5). Positiveness of transgenic plant was examined by PCR using plant DNA as a template, and an AtActin-2 gene was amplified as a control. Western blot was performed using GFP-Tag mouse antibody (Abmart) and plant β actin mouse monoclonal antibody (Cwbiotech).

### Measurement of seed size and weight per thousand seeds

Seeds from different transgenic lines were coated by gold dust, and pictures were taken under a scanning electron microscope. The length and width of 20 seeds were measured using ImageJ program. Weight per thousand seeds was obtained through weighing 1000 seeds.

### Quantitative analysis of Fatty Acid content

Seeds (10 mg) from Col-0 and transgenic lines, with four biological replicates, were used for extraction of fatty acids as previously described^38^. After extraction, the fatty acids were subjected to gas chromatography (GC2014, Shimadzu). Peaks corresponding to each FA species can be identified by FAME analytical standard (Cat. no. 18920-1AMP, Supelco).

### Quantitative reverse transcriptase-PCR (qRT-PCR)

According to the instructions, total RNA was isolated from siliques of Col-0 and the transgenic lines using TRIzol reagent (Tiangen). The reverse transcription was carried out using a first-strand cDNA synthesis kit (TransGen Biotech). The cDNA was then used as templates for qRT-PCR using SYBR qPCR mix (Toyobo). After reaction on a LightCycler480 System (Roche), the relative expression level of each sample was quantified using an internal control. The *UKN1 (Glyma12g02310*) gene and *AtACTIN7* gene were chosen as an internal control for soybean (*Glycine max*) and *Arabidopsis* system, respectively. All the qRT-PCR primers are listed in Table S2.

### Transactivation of target promoter by *GmDREBL* in tobacco leaves

Using Gateway^®^ technology (Invitrogen), the 3 kb sequences upstream from the ATG codons of *WRI1* were inserted into pGWB435 to generate promoter: *LUC* reporter constructs. The 35S:*GmDREBL* plasmid and the reporter plasmid were transformed into *A. tumefaciens* GV3101. The *Agrobacterium* strains were cultured in LB medium with spectinomycin and rifampicin at 28 °C. After culture, the harvested Agrobacterium were re-suspended in infiltration buffer containing 10 mM MES, 0.2 mM acetosyringone and 10 mM MgCl2 with pH5.7 until the OD600 concentration reached 1.0. Using a syringe without needle, equal amounts of various combined bacterial suspensions were injected into the young leaves of the five-week-old tobacco plants, and the plants were cultured at 24 °C for 3 days. Then the injected leaves were cut off, spread with 100 mM luciferin (Promega) and placed in darkness for 5 min. Then the LUC activity was detected with a low-light cooled charge-coupled device imaging apparatus (iXon; Andor Technology). At least six independent biological replicates were performed for each experiment.

### ChIP-PCR assay

ChIP assays were carried out according to a published protocol^39^ with modifications. Siliques (3 g) of the *GmDREBL*-*GFP*-transgenic lines and Col-0 were cross-linked in 1% formaldehyde and the chromatin was isolated based on the previous procedures^39^. Using GFP antibody (EarthOX), the DNA-protein complex was immunoprecipitated. The precipitated DNA was further subjected to quantitative PCR analysis.

### Gel-shift assay

The recombinant protein of maltose binding protein–GmDREBL was expressed in Escherichia coli BL21 using pMAL-c5x vector and purified from cells using Amylose Resin (NEB). The examined fragments in promoters of target genes were annealed by using synthesized oligonucleotides. The gel-shift assay was performed using a LightShift Chemiluminescent EMSA Kit (Thermo) according to the manufacturer’s instructions. The sequences of oligonucleotides are listed in Table S2.

### The expression of *GmWRI1 homologues*, *GmDREBL, GmABI3* and *GmABI5* in soybean transgenic hairy roots expressing the *GmDREBL, GmABI3* and *GmABI5*

pBI121-*GmDREBL*, pBI121-*GmABI3* and pBI121-*GmABI5* vectors were constructed and were transfected into *A. rhizogenes* K599 respectively. One-week-old seedlings of soybean Kefeng 1 were infected with K599 or K599 harboring the construct mentioned above following previous description^40^,^41^. Hairy roots were generated at the infection sites after 14 d and the seedlings were immersed in water for 3 d and then the original main roots were removed by cutting. Hairy roots of ~1 cm in length were collected and RNAs were extracted for further determination of *GmWRI1 homolgoues, GmDREBL, GmABI3* and *GmABI5* expression level. All PCR primers were listed in Table S2.

### Phylogenetic tree analysis of *DREBL* gene promoters from wild type and cultivated soybean

DNA was extracted from wild type and cultivated soybeans, and promoters of *GmDREBL* from cultivated soybean and promoters of *GsDREBL* from wild type soybean were cloned, sequenced and compared. Phylogenetic tree analysis was performed using PHYLIP (V. 3.69).

### Comparison of *DREBL* expression in cultivated soybean and wild type soybean

RNA was extracted from developing seeds of 90 cultivated and 69 wild type soybeans, and cDNA was made through reverse transcription. The *DREBL* expression was examined through real-time PCR.

## Additional Information

**How to cite this article**: Zhang, Y.-Q. *et al.* Soybean GmDREBL Increases Lipid Content in Seeds of Transgenic *Arabidopsis. Sci. Rep.* **6**, 34307; doi: 10.1038/srep34307 (2016).

## Supplementary Material

Supplementary Information

Supplementary Table S1

Supplementary Table S2

## Figures and Tables

**Table 1:** The relationship of variation types of *GmDREBL* promoters and oil content.

variation type	number of soybean accession	oil content (%)	SD	Position (bp)						
				−1823	−1442	−1324	−1120	−944	−404	−31
1	1	23.1		..TTTTTTATTTTTTTACTA……T	……GG	.GCATAA	TA	TTTGTCACCTGCCCC	GTTAGCATT	TTTT..A
2	51	18.2	3.20	..TTTTTTATTTTTTTACTAACTATAT	……GA	.GCATAA	..	……………	………	TTT…A
3	4	14.8	2.95	.TTTTTTTATTTTTTTACTA……T	……GG	.GCATAA	TA	……………	………	TTTT..A
4	4	13.0	1.34	.TTTATTTTTTTACTAACTA….TAT	……GG	.GCATAA	AA	……………	GTTAGCATT	TTTT..A
5	2	12.8	1.06	..TTTTTTATTTTCTTACTA……T	AGGTTCGG	.GTATAA	AA	……………	GTTAGCATT	TTTT..A
6	10	12.7	1.42	ATTTTTTTAATTTTTTACTAACTATAT	……GG	TGCATAT	AA	A…………..	GTTAGCATT	TTTTT.A
7	4	12.7	1.56	.TTTTTTTATTTTTTTACTAACTATAT	……GA	.GCATAA	..	……………	………	TTTT..A

Cultivated soybeans and wild soybeans of which their promoters clustered with cultivated soybeans have higher oil content compared to wild soybeans whose promoters clustered far away from the cultivated soybeans. Taking Williams 82 as reference sequence (variation type 2).
