# Supplementary material for: Soybean GmDREBL Increases Lipid Content in Seeds of Transgenic Arabidopsis
Source: Sci Rep. 2016 Oct 3;6:34307. doi: 10.1038/srep34307 (PMC5046110; doi:10.1038/srep34307)
Supplement: Supplementary Information [file srep34307-s1.pdf]

Soybean GmDREBL Increases Lipid Content in Seeds of Transgenic *Arabidopsis*

Running title: *GmDREBL* increases lipid content in seeds

Yu-Qin Zhang[1,2], Xiang Lu[1,2], Fei-Yi Zhao[3], Qing-Tian Li[1], Su-Ling Niu[1], Wei Wei[1], Wan-Ke Zhang[1], Biao Ma[1], Shou-Yi Chen[1]*, Jin-Song Zhang[1]*

[1] State Key Laboratory of Plant Genomics, Institute of Genetics and Developmental Biology, Chinese Academy of Sciences, Chaoyang District, Beichen West Road, Campus #1, No.2, Beijing 100101, China; [3]School of Bioengineering & Biotechnology, Tianshui Normal University, Tianshui, Gansu 741000, China

[2] Contribute equally to the work.

* To whom correspondence should be addressed. E-mail: sychen@genetics.ac.cn or jszhang@genetics.ac.cn

Figure S1

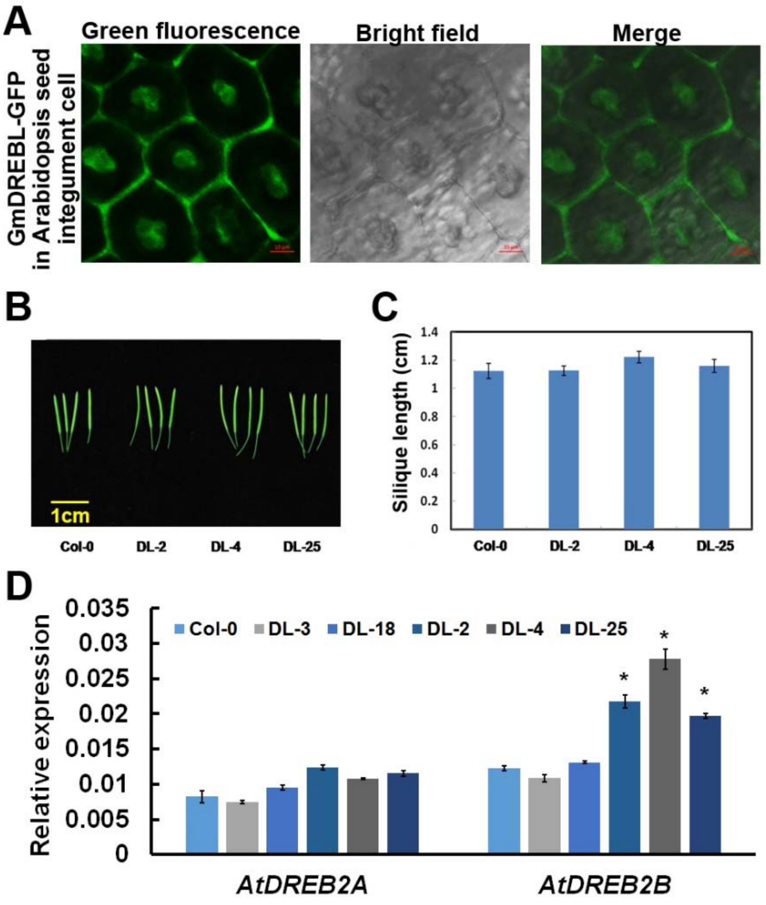

Figure S1 GmDREBL-GFP localization in *GmDREBL-GFP* transgenic Arabidopsis seeds and silique length comparison.

(A) GmDREBL-GFP localization in integument cells of *GmDREBL-GFP* transgenic Arabidopsis seeds. (B) The silique length of wild type Col-0 and transgenic plants with *GmDREBL-GFP*. The line stands for 1 cm. (C) The statistical result for the silique length of wild type Col-0 and transgenic plants with *GmDREBL-GFP*. Bars indicate SD (n=200). (D) The expression of *AtDREB2A* and *AtDREB2B* in *GmDREBL*-tragenic plants. * indicate significant difference (p-value < 0.05).

Figure S2

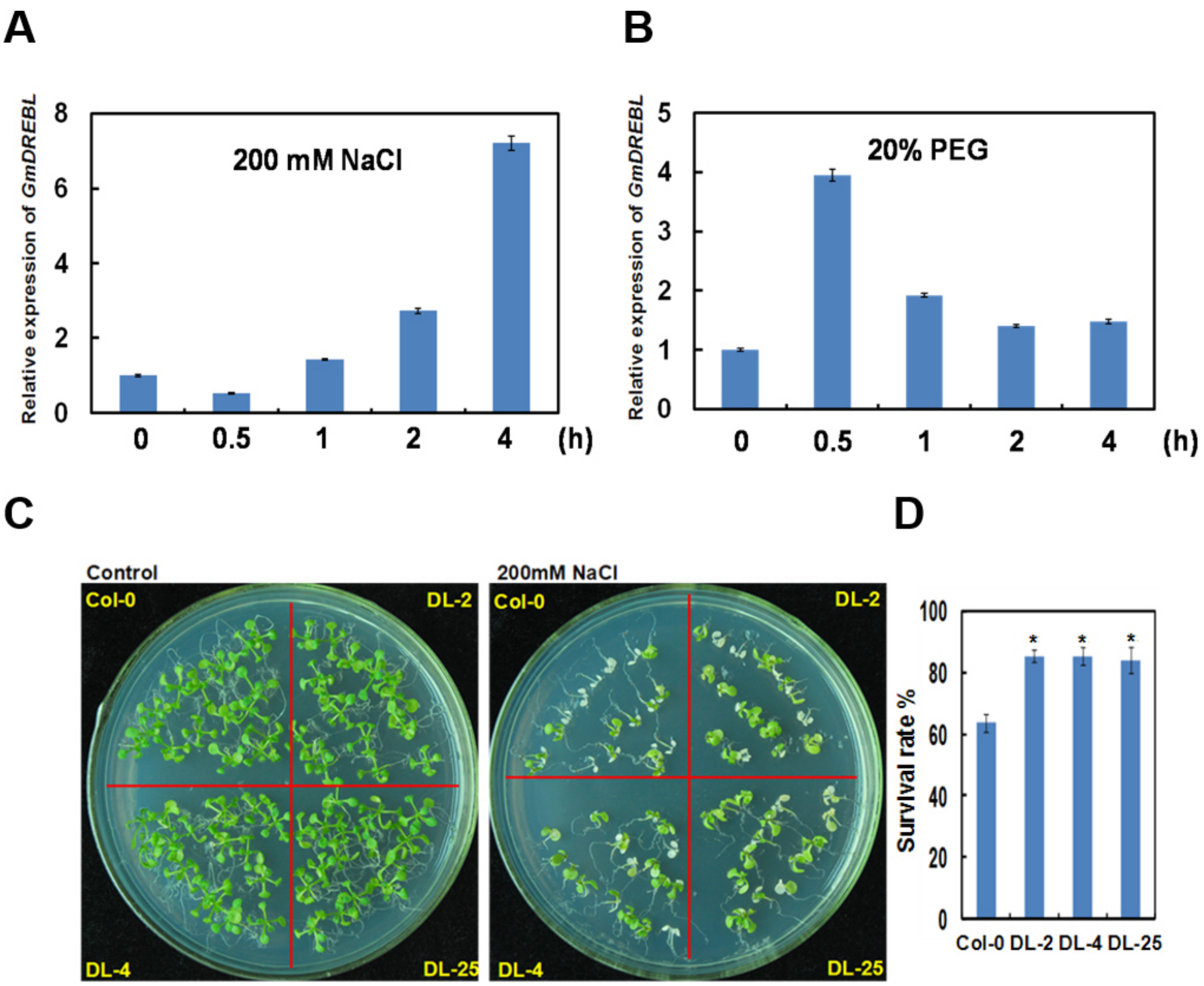

Figure S2 The expression pattern of *GmDREBL* under different stress treatments.

(A) The expression of *GmDREBL* under 200mM NaCl treatment. (B) The expression of *GmDREBL* under 20%PEG treatment. (C) The phenotype of Col-0 and *GmDREBL*-overexpressing plants under normal condition and 200 mM NaCl treatment. (D) The survival rate of Col-0 is lower than that of *GmDREBL*-overexpressing plants under 200 mM NaCl treatment. Bars indicate SD (n=68). * indicates significant difference ($P < 0.05$).

Figure S3

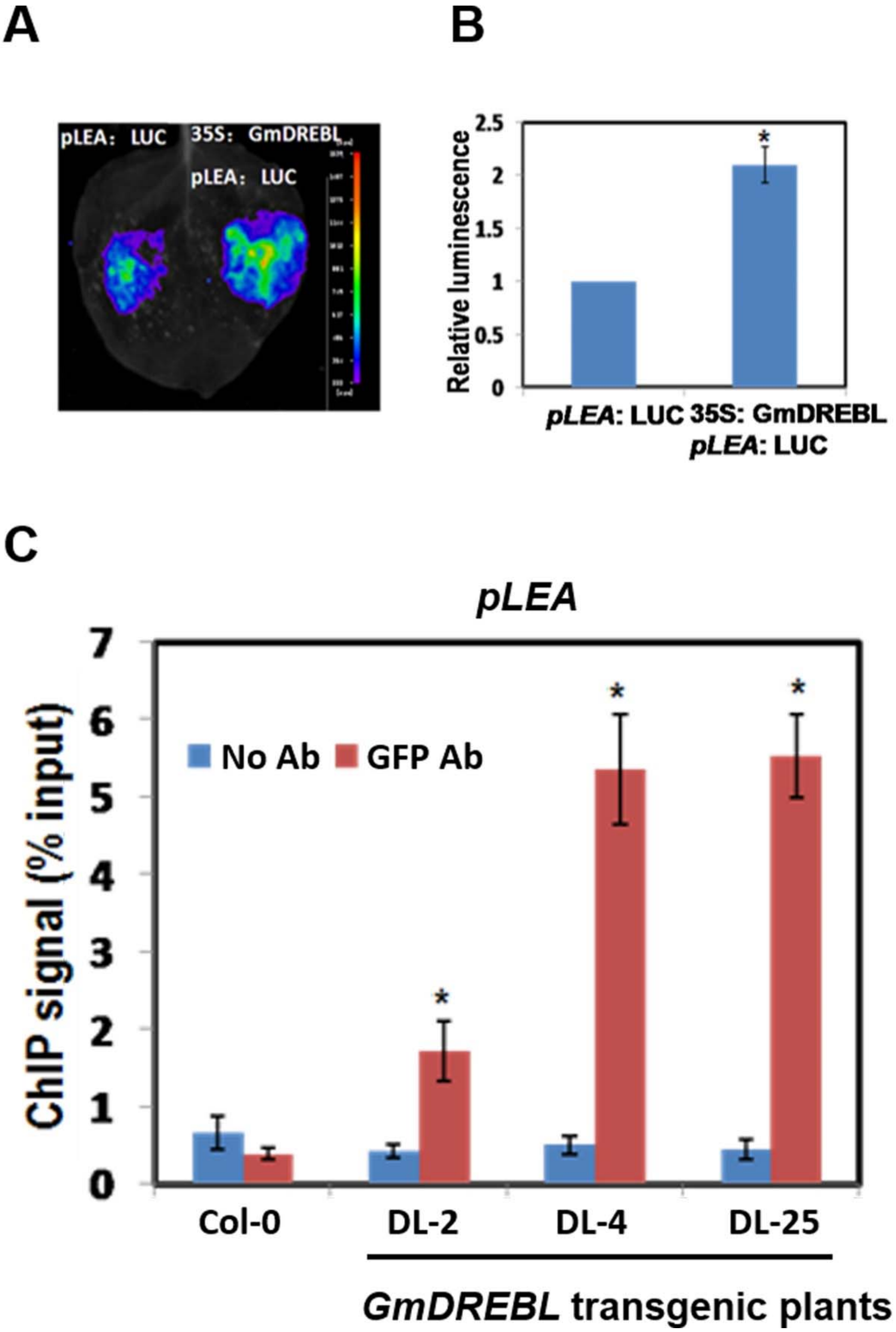

Figure S3 GmDREBL can directly bind to the promoter region of *LEA*.

(A) The LUC image of tobacco leaves co-infiltrated with GV3101 containing different combination of constructs. (B) Quantitative analysis of LUC luminescence intensity in (A). Asteriskes indicate significant differences compared with the control ($P<0.05$). (C) The intensity of ChIP signal of Col-0 plants and *GmDREBL*-overexpressing plants with a GFP tag for GmDREBL. Bars indicate SD (n=4). * indicate significant difference (p-value < 0.05).
